# Genomic analyses of germline and somatic variation in high-grade serous ovarian cancer

**DOI:** 10.1186/s13048-023-01234-x

**Published:** 2023-07-17

**Authors:** A. W. Adamson, Y. C. Ding, L. Steele, L. A. Leong, R. Morgan, M. T. Wakabayashi, E. S. Han, T. H. Dellinger, P. S. Lin, A. A. Hakim, S. Wilczynski, C. D. Warden, S. Tao, V. Bedell, M. C. Cristea, S. L. Neuhausen

**Affiliations:** 1 Department of Population Sciences, Beckman Research Institute of City of Hope, CA Duarte, USA; 2 Formerly, Department of Medical Oncology, City of Hope National Medical Center, Duarte, CA USA; 3 Currently at Regeneron Pharmaceuticals Inc, Formerly City of Hope National Medical Center, Duarte, CA USA; 4 Formerly, Department of Surgery, City of Hope National Medical Center, Duarte, CA USA; 5 Department of Surgery, City of Hope National Medical Center, Duarte, CA USA; 6 Department of Pathology, City of Hope National Medical Center, Duarte, CA USA; 7 Integrative Genomics Core, Beckman Research Institute of City of Hope, Duarte, CA USA; 8 Cytogenetics Core, City of Hope National Medical Center, Duarte, CA USA

**Keywords:** High-grade serous ovarian cancer, Homologous recombination repair, Germline mutations, Somatic mutations, Somatic copy number alterations

## Abstract

**Background:**

High-grade serous ovarian cancers (HGSCs) display a high degree of complex genetic alterations. In this study, we identified germline and somatic genetic alterations in HGSC and their association with relapse-free and overall survival. Using a targeted capture of 557 genes involved in DNA damage response and PI3K/AKT/mTOR pathways, we conducted next-generation sequencing of DNA from matched blood and tumor tissue from 71 HGSC participants. In addition, we performed the OncoScan assay on tumor DNA from 61 participants to examine somatic copy number alterations (SCNA).

**Results:**

Approximately one-third of tumors had loss-of-function (LOF) germline (18/71, 25.4%) or somatic (7/71, 9.9%) variants in the DNA homologous recombination repair pathway genes *BRCA1*, *BRCA2*, *CHEK2*, *MRE11A*, *BLM,* and *PALB2*. LOF germline variants also were identified in other Fanconi anemia genes and in MAPK and PI3K/AKT/mTOR pathway genes. Most tumors harbored somatic *TP53* variants (65/71, 91.5%)*.* Using the OncoScan assay on tumor DNA from 61 participants, we identified focal homozygous deletions in *BRCA1*, *BRCA2*, *MAP2K4*, *PTEN*, *RB1*, *SLX4*, *STK11*, *CREBBP*, and *NF1.* In total, 38% (27/71) of HGSC patients harbored pathogenic variants in DNA homologous recombination repair genes. For patients with multiple tissues from the primary debulking or from multiple surgeries, the somatic mutations were maintained with few newly acquired point mutations suggesting that tumor evolution was not through somatic mutations. There was a significant association of LOF variants in homologous recombination repair pathway genes and high-amplitude somatic copy number alterations. Using GISTIC analysis, we identified *NOTCH3, ZNF536*, and *PIK3R2* in these regions that were significantly associated with an increase in cancer recurrence and a reduction in overall survival.

**Conclusions:**

From 71 patients with HGCS, we performed targeted germline and tumor sequencing and provided a comprehensive analysis of these 557 genes. We identified germline and somatic genetic alterations including somatic copy number alterations and analyzed their associations with relapse-free and overall survival. This single-site long-term follow-up study provides additional information on genetic alterations related to occurrence and outcome of HGSC. Our findings suggest that targeted treatments based on both variant and SCNA profile potentially could improve relapse-free and overall survival.

**Supplementary Information:**

The online version contains supplementary material available at 10.1186/s13048-023-01234-x.

## Background

High-grade serous ovarian cancer (HGSC) is the most common ovarian cancer histologic subtype with the majority of patients diagnosed at a late stage [1]. The standard treatment for HGSC involves both surgical cytoreduction and systemic paclitaxel and platinum chemotherapy with increasing use of targeted treatments of bevacizumab and/or poly ADP-ribose polymerase inhibitors (PARP-i) [2–4]. Despite aggressive frontline therapy, many patients with ovarian cancer eventually relapse, resulting in only 20–30% survival after five years [5]. These dismal statistics underscore the need for earlier detection and new treatments following diagnosis.

HGSC originates in the fallopian tube [6, 7] and is characterized by early acquisition of *TP53* variants [8, 9]. Other characteristics are extensive somatic copy number alterations (SCNA) [9, 10] with few somatic point variants [11] and inactivation of DNA homologous recombination repair (HRR) primarily through germline and somatic variants of *BRCA1* and *BRCA2* [9]. Although less frequent, variants in additional Fanconi anemia pathway genes that play key roles in HRR (e.g., *BRIP1*, *PALB2, RAD51C/D*), and *CHEK2*, a DNA damage response gene, have been associated with HGSC [12–15]. Amplification of regions containing the oncogenes *CCNE1, MECOM*, and *MYC* and deletions of regions containing the tumor suppressor genes *RB1*, *NF1*, and *PTEN* are commonly observed [9, 16, 17]. An integrated analysis combining variant data, copy number changes, and changes in gene expression determined that the main pathways altered in HGSC are the HRR, RB1, PI3K/AKT/mTOR, and NOTCH signaling pathways [9].

In this study, we identified and characterized germline and somatic genetic alterations in HGSC and their associations with relapse-free and overall survival. From paired germline and tumor DNA samples from 71 HGSC patients treated at City of Hope (COH), we conducted targeted sequencing of 557 genes in pathways involved in response to DNA damage, DNA repair, cell-cycle regulation, programmed cell death, MAPK, and PI3K/AKT/mTOR signaling including known drivers in HGSC tumorigenesis [18]. In addition, we used the OncoScan assay to determine genome-wide SCNAs and loss of heterozygosity (LOH) in 61 primary ovarian tumors.

## Results

### Clinical/demographic data

Participants were 71 women with Stage III or IV HGSC with matching germline and tumor samples (Table 1). The median age at diagnosis of cancer was 58 years and median survival was 37.8 months (Table 1). A total of 114 tumors were examined, comprising a mixture of primary debulking, metastatic, and recurrent tumors, often from multiple sites (details in Supplemental Table 1).

**Table 1:** Clinical characteristics of the 71 ovarian cancer cases

	Category	No. (%)
Race and ethnicity	Non-Hispanic White	55 (77.5)
	Hispanic/Latina	11 (15.5)
	Asian	5 (7.0)
Median age of diagnosis (years)	58	
Age at diagnosis (years)	< 50	7 (9.9)
	50–69	53 (74.6)
	≥ 70	11 (15.5)
Median overall survival (months)	37.8	
Median RFS^a^ (months)	16.5	
Median follow-up^b^ (months)	55.8	
Stage	IIIA-IIIC	62 (87.3)
	IV	9 (12.7)
Debulking status	Optimal	61 (85.9)
	Suboptimal	7 (9.9)
	Unable to debulk	1 (1.4)
	Unknown	2 (2.8)
Chemotherapy therapy	Adjuvant	57 (80.3)
	Neoadjuvant	14 (19.7)
Chemotherapy delivery	Intraperitoneal	28 (39.4)
	Intravenous	43 (60.6)
Tumors investigated	Primary	64
	Metastatic	50
Tumors per patient	1	44
	2	17
	≥ 3	10

^a^Relapse-free survival (RFS) as of 6/22/21

^b^Time to first recurrence from last chemo dose. No recurrence as of 06/22/21

### Germline and somatic variant identification by pathway

#### HRR genes

**Fig. 1:**
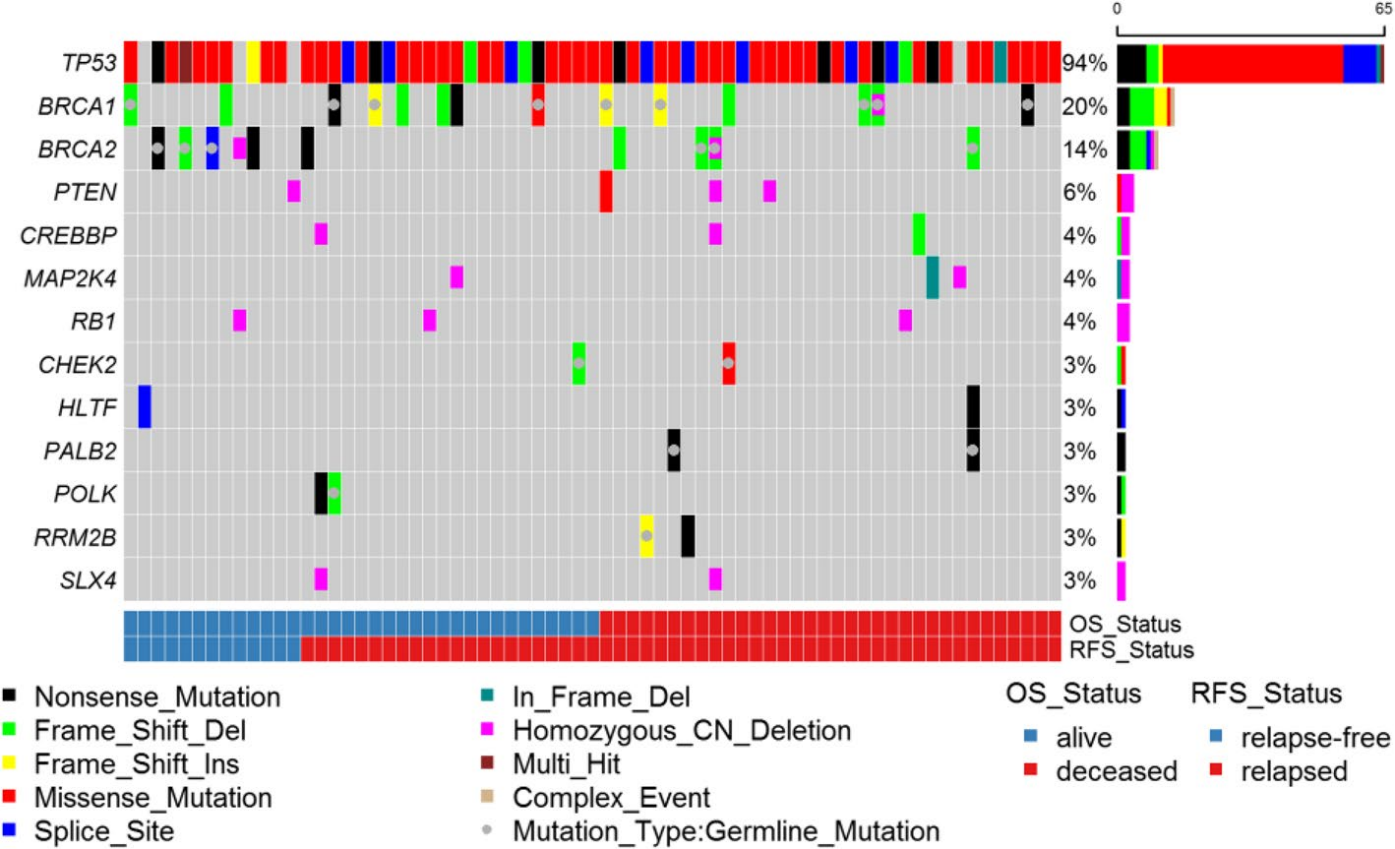
Oncoplot of LOF germline mutations and non-silent somatic mutations. Only genes with variants in at least 3% of patients are displayed. OS = overall survival. RFS = relapse-free survival

Consistent with previous studies of HGSC [9], we observed germline loss-of-function (LOF) variants in 17 patients (23.9%) in known ovarian cancer HRR predisposition genes including nine (12.7%) in *BRCA1*, six (8.5%) in *BRCA2*, two (2.8%) in *PALB2*, and one in *MRE11A* (1.4%) (Fig. 1). Patient #56 had germline frame-shift deletions in both *BRCA2* and *PALB2* with LOH of *BRCA2* in the somatic tissue. For patients with germline LOF variants, we checked for LOH in the corresponding tumor. Similar to the HGSC data in The Cancer Genome Atlas (TCGA) [19], we observed a high frequency of LOH with 6/9 germline truncation variants in *BRCA1*, 5/6 in *BRCA2*, 1/2 in *PALB2*, and 1/1 in *MRE11A* (Supplemental Table 3). We identified somatic LOF *BRCA1* variants in five (7.0%) tumors, *BRCA2* in three (4.2%) tumors, and *BLM* in one tumor (1.4%) (Fig. 1). From our OncoScan analysis, we observed two somatic homozygous deletions in *BRCA2*, one in *BRCA1,* and two in *SLX4* (pink half bar in Fig. 1). Of note, patient #41 carried the H/L germline founder *BRCA1* exon 9–12 deletion and had lost the second copy of *BRCA1* as seen from Oncoscan analysis. Patient #14 had a germline LOF *BRCA2* frame-shift deletion and the OncoScan analysis detected that the tumor had homozygous deletions of *BRCA2* and *SLX4*. In total, 38% (27/71) of HGSC tumors harbored LOF variants in HRR genes. Additionally, a *BRIP1* germline missense variant (P47A) in patient #45 is potentially pathogenic (Supplemental Table 3).

#### Other Fanconi anemia and DNA damage response genes

In Fanconi anemia pathway genes nominally associated with HGSC, we identified germline LOF variants in *FAN1*, *FANCD2*, *FANCI*, and *FANCL* (Supplemental Fig. 1). For known ovarian cancer genes involved in cell cycle regulation, as expected, we found a high frequency of somatic *TP53* variants (65/71, 91.5%) (Fig. 1, Supplemental Table 4) [8, 9]*.* We identified a germline *CHEK2* frameshift deletion LOF variant in patient #67 with LOH in the tumor and the LOF I157T missense variant in patient #15. From the OncoScan analysis, we identified three somatic homozygous deletions in *RB1* (Fig. 1). In DNA damage repair genes nominally or not known to be associated with HGSC, we identified germline LOF variants in cell cycle (*MCPH1* and *PKMYT1),* double-strand break (*EME2*, *POLQ*, *RECQL5*, and *SPO11*), nucleotide excision repair (*ERCC2* and *POLK*), base excision repair (*NEIL3* and *POLG*), mismatch repair (*EXO1*), and post-replication repair (*RAD18*) pathways. Somatic LOF variants involved in cell cycle (*RBL2* and *TFDP1*), mismatch repair (*PMS1* and *RFC4*), nucleotide excision repair (*POLK*), and base excision repair (*POLL*) also were identified (Supplemental Fig. 1).

#### MAPK and PI3K/AKT/mTOR pathway genes

From the combined OncoScan and sequence analyses, we observed somatic variants in MAPK-pathway genes with one in-frame deletion and two homozygous deletions in *MAP2K4* and one homozygous deletion in *NF1* (Fig. 1 and Supplemental Fig. 1). We observed germline LOF variants in the PI3K/AKT/mTOR genes *FKBP11*, *FKBP7*, and *IFNA5* along with a somatic LOF variant in *PIK3R1* (Supplemental Fig. 1). We also identified three somatic homozygous deletions and one somatic missense variant in *PTEN*, two somatic homozygous deletions and one somatic frameshift deletion in *CREBBP* and a somatic hotspot missense variant in *PIK3CA* (Fig. 1 and Supplemental Fig. 1).

### Analysis of multiple tumor samples from the same patient

For the 12 patients with multiple samples from the primary debulking including metastatic sites, the somatic variants were the same except in three patients where one of the multiple tumors from the primary debulking acquired a somatic mutation (Supplemental Table 4). Similarly, for the 16 patients with samples from more than one debulking surgery, no newly acquired somatic variants were observed with a few exceptions (Supplemental Table 4), none of which have been associated with disease progression or response to therapy.

### SCNA results

**Fig. 2:**
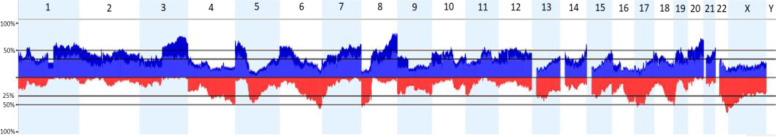
Overall frequency of SCNAs in 61 primary HGSC tumors. From Nexus Copy Number v. 10, red indicates regions of loss, blue indicates regions of gain, and dark blue indicates regions of high-copy gain

**Fig. 3:**
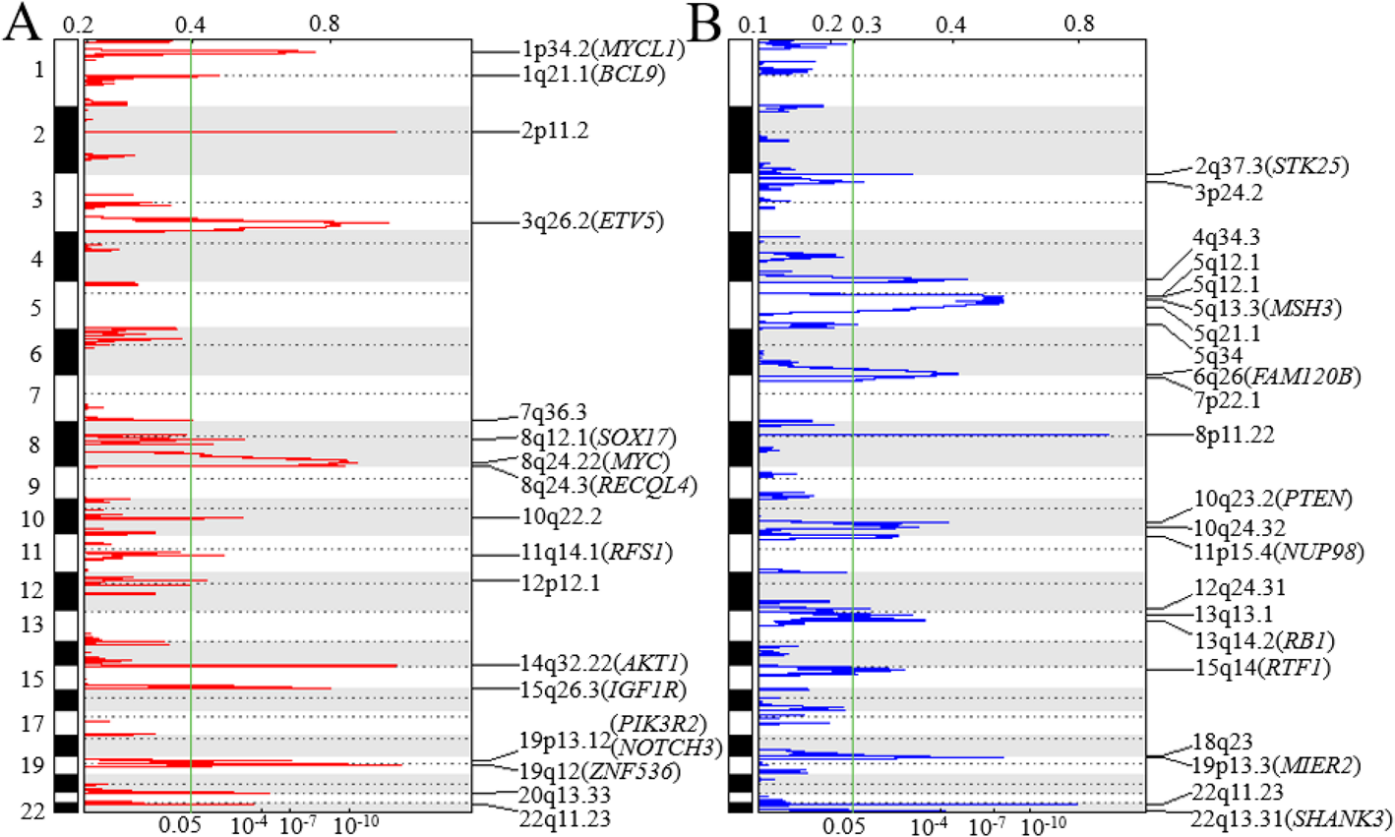
Significantly amplified (panel **A**) or deleted (panel **B**) regions of genome. GISTIC analysis was performed to identify significant recurrent focal SCNAs in 61 tumor samples. Genomic positions of SCNA regions are oriented vertically. GISTIC q value and G scores are shown at the bottom and top, respectively. The green line marks GISTIC q value of 0.05. Potential candidate or common cancer genes for each SCNA region are included in the parenthesis next to each SCNA region

A genome-wide view of the SCNAs in the 61 tumors is shown in Fig. 2. Using Nexus Copy V10, the most frequent broad-arm levels of amplification were chromosomal regions 1q, 3q, 5p, 6p, 7q, 8q, and 20q and the most frequent regions of loss were chromosomal regions 4q, 6q, 8p, 17p, 18q, and 22q. There were 17 regions of amplification on 13 chromosomes and 22 regions of deletion on 16 chromosomes with GISTIC q-value less than 0.05 (Fig. 3). The chromosomal locations, peak limits, and q-values for the 39 regions are listed in Supplemental Table 5. The five most significant regions of amplification were at 19q12 (q = 1.06E-11), 14q32.33 (q = 3.07E-11), 2p11.2 (q = 3.43E-11), 3q26.2 (q = 1.48E-10), and 8q24.21 (q = 2.80E-8). Within these regions are oncogenes *CCNE1* in 19q12, *MECOM* in 3q26.2, *MYC* in 8q24.21, and *MYCL1* in 1p34.2 (q = 7.75E-6). The most significant (q < 0.01) SCNA losses were at 6q26 (q = 1.45E-5; containing *MAP3K4* and *ARID1B*), 7p22.1 (q = 1.12E-3; containing *PMS2)*, 5q21.1 (q = 1.31E-3; containing *APC)* 10q23.31 (q = 1.61E-3; containing *PTEN)*, 13q14.2 (q = 3.29E-3; containing *RB1*), and 5q13.1 (q = 4.27E-3; containing *PIK3R1*).

### Association of LOF in core HRR genes and incidence of high-amplitude SCNAs

In the 61 samples with both sequence and SCNA data from OncoScan, there were LOF variants in the core HRR genes *BRCA1*, *BRCA2*, *PALB2*, in 19 patients (Supplemental Tables 3 and 4). High amplitude SCNAs, in 169 chromosomal regions containing 2369 genes, found in more than 5% samples were included in the test of association between LOF in *BRCA1, BRCA2*, and *PALB2* and incidence of SCNAs in each gene. We found statistically significant associations [*P* < 0.05 and false discovery rate (FDR) < 0.25] of 262 genes in 10 chromosomal regions (6p25.2, 6p25.3, 8p23.1, 8q23.3, 8q24.13, 8q24.21, 8q24.22, 8q24.23, 8q24.3, 15q26.3) (Supplemental Table 6). All 262 genes had odds ratios (OR) greater than 1.0 (95% confidence interval not including 1.0), indicating that LOF variants in these three genes were associated with increased incidence of high-amplitude SCNAs. The 262 genes were enriched (adjusted *P* < 0.05) in pathways involved in immune responses and biological functions related to transcriptional regulation (Supplemental Table 7).

From analysis of data from 356 HGSC tumor samples in TCGA, 24 had LOF variants in *BRCA1* (16 germline, 8 somatic), 25 had LOF variants in *BRCA2* (19 germline, 6 somatic), and 2 had somatic LOF variants in *PALB2* for a total of 51 patients (14%). Of the 262 SCNA genes significantly associated with variants in one of the three genes, 168 genes had been measured by the Affymetrix SNP 6.0 array in TCGA and had high-amplitude SNCAs in at least 5% of the 356 TCGA samples. For 136 of the 168 genes, the association was validated (*P* < 0.05 and FDR < 0.25, and consistent direction of OR > 1.0) (Supplemental Table 6); the remaining 36 genes showed a consistent OR (point estimation of OR greater than 1.0) but were not significant at *P* < 0.05 and FDR < 0.25.

### Association of LOF variants in *BRCA1*, *BRCA2*, and *PALB2* and relapse-free and overall survival

We tested the association of LOF variants in the high-penetrant genes, *BRCA1*, *BRCA2*, and *PALB2,* on overall and relapse-free survival after definitive surgery and adjuvant therapy. For the 61 COH patients with both sequencing and SCNA data, we found 19 LOF variants including 12 germline variants (6 in *BRCA1*, 5 in *BRCA2,* 1 in *PALB2*) and 7 somatic variants (5 in *BRCA1*, 2 in *BRCA2*). From TCGA ovarian cancer data, a total of 51 LOF germline or somatic variants (24 in *BRCA1*, 25 in *BRCA2*, and 2 in *PALB2*) were observed in 356 patients. Compared to patients not carrying LOF variants*,* 19 of 61 COH (panel A) and 51 of 356 TCGA patients (panel C) carrying LOF variants showed significantly (*p* < 0.05) better relapse-free survival (Supplemental Fig. 2). For overall survival, highly significant overall survival advantage was observed in TCGA patients with LOF variants (panel D), but not in COH patients (panel B), likely due to small sample size (Supplemental Fig. 2).

### Association of SCNAs with relapse-free and overall survival

**Fig. 4:**
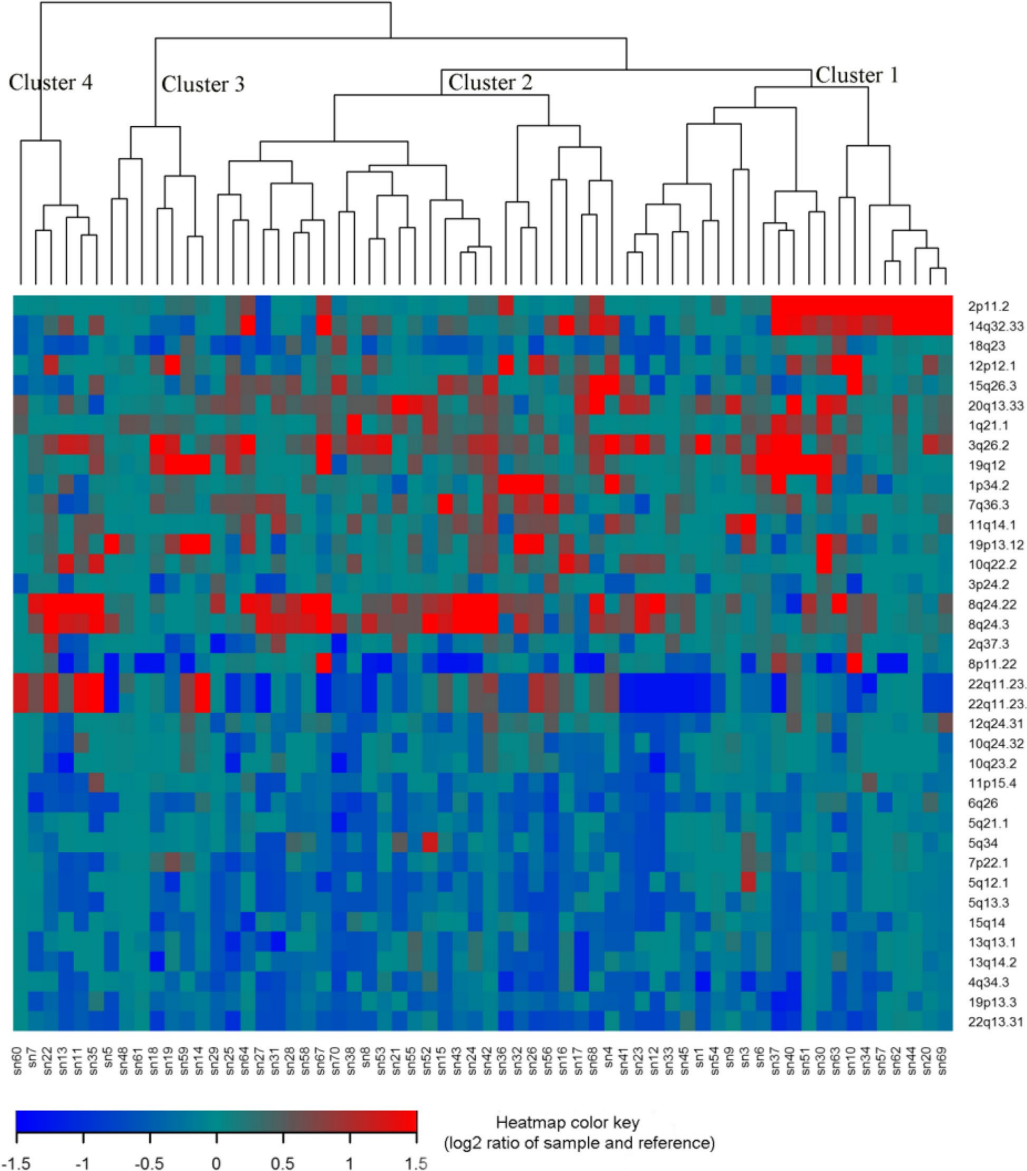
Unsupervised hierarchical clustering of 61 primary HGSC samples using Oncoscan data from 37 SCNAs. Changes in copy number for 37 SCNAs with GISTIC residual q value < 0.05 were used to perform unsupervised hierarchical clustering of 61 primary HGSCs samples. Each column represents a tumor sample and rows represent the 37 SCNAs. As shown in the color key, genomic regions of gain are represented in red and regions of loss are represented in blue

**Fig. 5:**
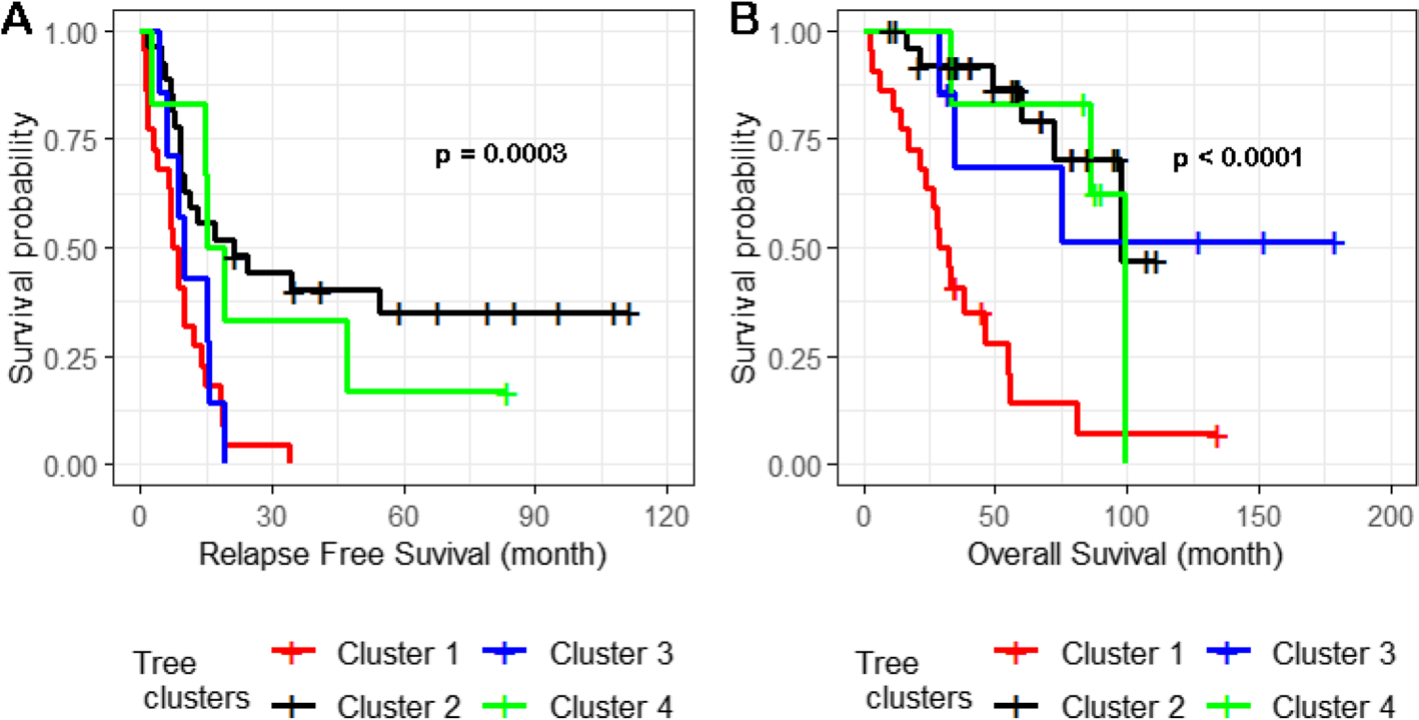
Relapse-free (**A**) and overall survival analysis (**B**) of four clusters of patients defined by SCNAs. Kaplan–Meier analysis was used to evaluate the difference in survival for COH patients in the four clusters of the unsupervised hierarchical tree in Fig. 4

For the 61 primary HGSC tumors, we applied an unsupervised hierarchical clustering algorithm to the 37 of 39 recurrent SCNAs with residual (after removing segments shared with higher peaks) q values less than 0.05 and identified four subgroups of patients in the dendrogram (Fig. 4). In a Kaplan–Meier analysis, the four subgroups had highly significant differences in both relapse-free surivival (log-rank test *P* = 0.0003) (Fig. 5A) and overall survival (log-rank test *p* < 0.0001) (Fig. 5B).

**Fig. 6:**
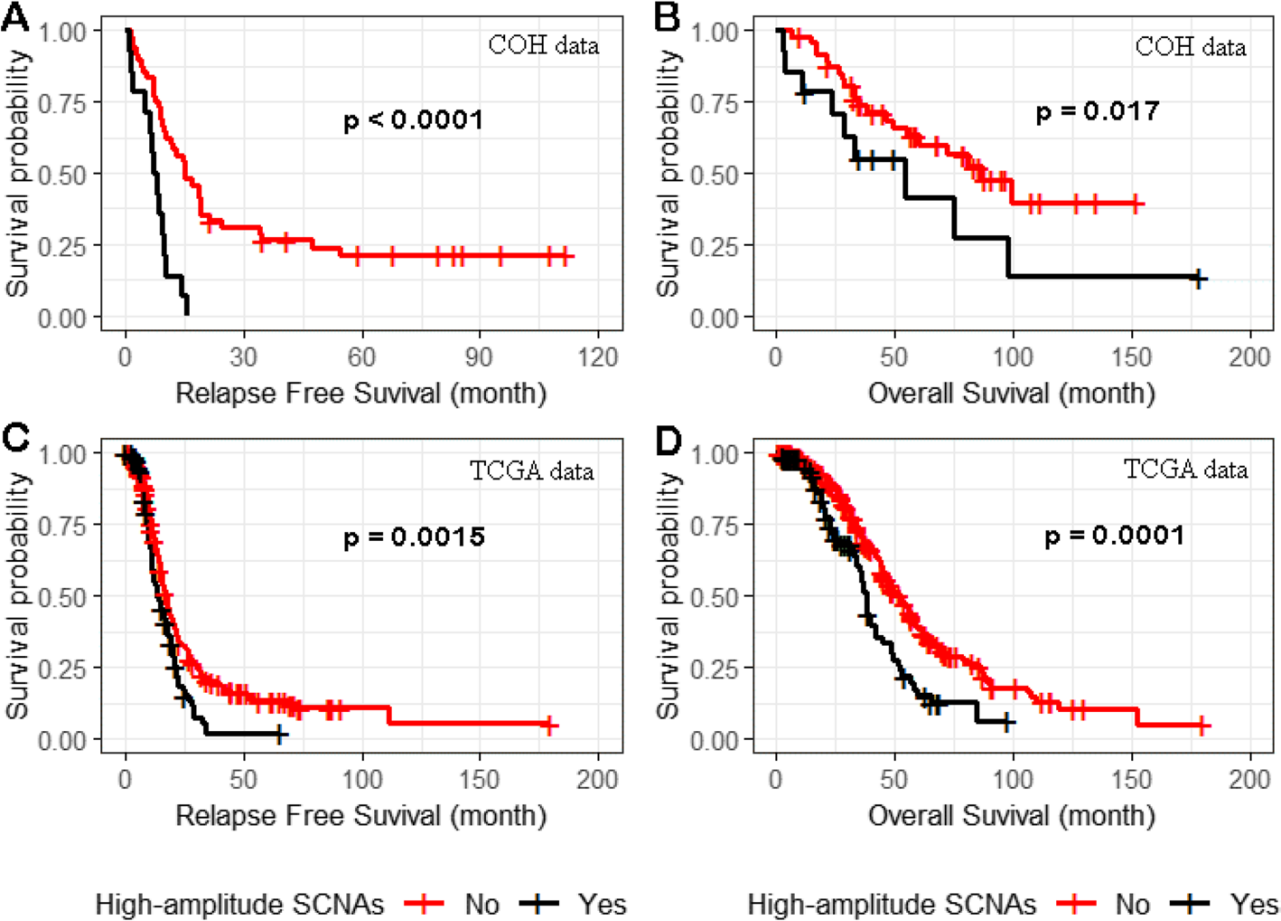
Relapse-free and overall survival related to high-amplitude SCNAs in *NOTCH3, ZNF536*, and *PIK3R2*. Compared to patients not carrying high copy number gains in *NOTCH3, ZNF536*, and *PIK3R2*, patients from both COH (panels **A** and **B**) and TCGA (panels **C** and **D**) carrying high copy gains in at least one of the three genes showed significantly worse relapse-free survival (panel **A** and **C**) and overall survival (panel **B** and **D**), respectively

For high-amplitude SCNAs defined as regions of amplification with ≥ 4 copies or homozygous deletions and where a minimum of 5 of 61 tumors carried a high-amplitude SCNA in the region, we tested the association of SCNA and relapse-free survival and overall survival in 13 of the 17 regions of SCNAs gain and 7 of 22 regions of SCNA loss. High-amplitude SCNA gains in 4 of the 13 regions (3q26.2, 15q26.3, 19p13.12, and 19q12) showed evidence (*p* < 0.05) of association with a reduction in relapse-free survival (Supplemental Table 8); associations of two (19q12 and 19p13.12) of four regions were validated (*p* < 0.05) in TCGA data (Table 2). In the 19q12 region, only *ZNF536* had a significant association (*p* = 0.014). In the 19p13.12 region, *NOTCH3*, *ILVBL*, *SYDE1*, and *PIK3R2* had *p* < 0.05. *NOTCH3*, *ILVBL*, and *SYDE1* shared the same SCNAs distribution across 8 of 61 samples (*p* value = 0.047); therefore, we selected *NOTCH3* as the representative in the following combined analysis of SCNAs in the two validated regions; *NOTCH3* has been reported to be a potential oncogene [7]. Compared to patients not carrying high-amplitude gains in *ZNF536, PIK3R2,* and *NOTCH3,* patients from both COH (Fig. 6A and B) and TCGA (Fig. 6C and D) carrying high copy gains in at least one of the three genes showed significantly worse relapse-free survival (Fig. 6A and C, *p* < 0.01) and worse overall survival (Fig. 6B and D, *p* < 0.05). Homozygous deletions in the seven regions of SCNA loss were not associated with relapse-free or overall survival (Supplemental Table 9).

**Table 2:** Association between genes with high-amplitude SCNAs and relapse-free survival

		COH 61 samples			TCGA 356 samples		
Gene	Region	Frequency^a^	HR (95%CI)	*P*	Frequency^a^	HR (95%CI)	*P*
*NOTCH3*	19p13.12	13.11%	2.26 (1.01—5.03)	0.047	12.10%	1.67 (1.14—2.39)	0.01
*ZNF536*	19q12	9.84%	3.14 (1.26—7.86)	0.014	15.70%	1.44 (1.02 -2.00)	0.041
*PIK3R2*	19p13.11	8.19%	2.95 (1.13—7.73)	0.028	5.10%	1.84 (1.01 -3.14)	0.046

^a^*Frequency *Percentage of samples carrying high amplitude SCNAs, *HR* Hazard ratio, *CI* Confidence interval

We also investigated association of relapse-free and overall survival with the total percentage of genome changes in SCNA. Higher SCNA load was associated with better survival, but the evidence of association was only marginally significant or insignificant in patients from the COH study and from TCGA study, respectively (Supplemental Fig. 3).

## Discussion

All germline and somatic genetic variants identified in the 557 sequenced genes are provided in Supplemental Table 10. Consistent with previous studies of HGSC, we observed germline LOF variants in high- and moderate-penetrance HRR pathway genes [9, 12, 15, 19, 20]. In addition to the germline LOF variants in the HRR genes, *BRCA1*, *BRCA2*, *MRE11A*, and *PALB2*, LOF somatic variants in *BRCA1* and *BRCA2* were identified including homozygous deletions in *BRCA1* and *BRCA2*. From the combined sequencing and SCNA analysis, we found that approximately one third of the HGSC tumors harbored LOF variants in HRR genes and treatment with PARP inhibitors would be warranted [3].

We also observed LOF variants in Fanconi anemia pathway genes nominally associated with HGSC [9, 19]. These variants included germline LOF variants in *FAN1*, *FANCD2*, *FANCI*, and *FANCL*. Platinum-based therapies cause interstrand cross links (ICLs) and the Fanconi anemia pathway is critical for resolving ICLs for DNA repair through the HRR pathway [21]. Therefore, variants in Fanconi anemia genes may affect the efficacy of treatment and resultant survival. Interestingly, we observed a somatic nonsense variant in *BLM/RECQL3* which codes for a RecQ family DNA helicase, whose deficiency results in Bloom’s syndrome which is characterized by a predisposition to early development of multiple forms of cancer [22]. *BLM* has been shown to play a critical role in mitosis and multiple steps of HRR-dependent DNA repair [23]. While several germline LOF variants in *BLM* have been observed in HGSC tumors [19], only one somatic LOF variant was listed in the COSMIC (Catalogue of Somatic Mutations in Cancer) database [24].

Additional DNA repair pathway variants included germline LOF variants in double-strand break repair (*EME2*, *POLQ*, *RECQL5*, and *SPO11*), nucleotide excision repair (*ERCC2* and *POLK*), base excision repair (*NEIL3* and *POLG*), mismatch repair (*EXO1*), and post-replication repair (*RAD18*) pathways. Although uncommon, all these genes have documented germline protein truncating variants from other HGSC studies [19, 25, 26]. Of note, the identical germline splice site variant in *EXO1* and the frameshift deletions in *ERCC2*, *POLK*, and *POLQ* were reported in previous HGSC studies. We also observed somatic LOF variants in other DNA repair pathway genes with one each in *PMS1* (mismatch repair)*, POLK* (nucleotide excision repair), *POLL* (base excision repair), and *RFC4* (mismatch repair). A protein-truncating variant in *RFC4* was listed in COSMIC HGSC tumors whereas only missense variants were listed in *PMS1*, *POLL*, and *POLK* [24].

Alteration of the PI3K/AKT/mTOR pathway is a common occurrence in HGSC [9] and our results support this finding. We observed germline LOF variants in PI3K/AKT/mTOR pathway genes *FKBP11*, *FKBP7*, and *IFNA5*; in previous HGCS genomic studies, germline LOF variants were reported in *IFNA5* and *FKBP7*, but not *FKBP11* [19, 25]. Somatic variants in PI3K/AKT/mTOR pathways genes have been reported in HGSCs [9, 19, 27] and we identified LOF variants in *CREBBP* and *PIK3R1*. Our OncoScan analysis identified three somatic homozygous deletions in *PTEN* and two in *CREBBP*. In addition, we observed one somatic missense variant in *PIK3CA*, one in *CREBBP,* and one in *PTEN,* classified as pathogenic, reinforcing the importance of alteration in the PI3K/AKT/mTOR pathway in HGSC.

We observed variants in several genes involved in cell cycle regulation. As expected, we found a high frequency of somatic *TP53* variants [8, 9]*.* We also observed a germline LOF variant and a potentially pathogenic germline missense variant in the known ovarian cancer gene *CHEK2* [15, 19]. Our OncoScan analysis identified three somatic homozygous deletions in *RB1* consistent with a previous report that it was a recurrently mutated gene in HGSC [9]*.*

We observed somatic homozygous deletions in the MAPK pathway genes *MAP2K4* and *NF1*. The MAPK signaling pathway regulates many cellular processes, including gene expression, cell cycle, cell survival, cell death, and cell movement [28]. *MAP2K4* is listed in COSMIC as potentially having a role in both tumor suppression and as an oncogene [24]. Supporting its role as a tumor suppressor, homozygous focal deletions in *MAP2K4* have been identified in ovarian and breast cancers and it is suggested that loss of *MAP2K4* could alter function of the JNK (c-Jun N-terminal kinase) pathway [29, 30]. In addition, somatic copy number loss of *NF1* is common in HGSC [31, 32]. The NF1 protein has been found to play a role in regulating several intracellular processes and importantly is essential for reducing oncogenic Ras activity, supporting the hypothesis that it acts as a tumor suppressor [33].

For patients where we had multiple tissues from the primary debulking or tissue from multiple surgeries, in the secondary tumor sites, the somatic point mutations were maintained and there were few newly acquired somatic point mutations suggesting that tumor evolution was not through acquisition of somatic mutations in genes in the pathways we studied. This is consistent with prior reports that gene amplifications rather than somatic point mutations are observed in disease progression [34, 35]. We were not able to perform OncoScan analysis for multiple tissues from the same patient.

Along with frequent *TP53* variants, a hallmark of HGSC is profound genomic instability [9]. We identified several amplified regions containing driver oncogenes previously observed in HGSC including *CCNE1*, *MECOM*, *MYC*, and *MYCL1* and deletion regions containing tumor suppressor genes *RB1* and *PTEN* [9, 16, 17, 34, 36–38]. GISTIC identified several regions that, although CNAs, are unlikely to be somatic as the genomic regions they encompass include germline copy number variation (Supplemental Table 5). We only analyzed tumor tissue and so could not distinguish germline from somatic CNAs. These regions include the amplifications regions 14q32.33, 2p11.2, and 2p11.2 and the deletion regions 8p11.22, 22q11.23, and 4q34.3. Regardless of CNA source, they may affect outcome similar to LOF variants regardless of whether germline or somatic.

Since cancer-driver SCNAs tend to be shorter in length and higher in amplitude than passenger SCNAs [39], we sought to identify genes within high-amplitude SCNAs that were associated with relapse-free and overall survival. We determined that amplifications in *NOTCH3*, *ZNF536*, and *PIK3R2* were significantly (*P* < 0.05) associated with shorter relapse-free survival in both our data and TCGA data after adjusting for copy number mutation rate, age at diagnosis, and tumor stage. Debulking status (optimal versus suboptimal) and intra-peritoneal chemotherapy status (yes versus no) were not available in TCGA data. When we included these two variables in our data, the association remained significant for *NOTCH3* (*P* = 0.033) and *ZNF536* (*P* = 0.012), but not for *PIK3R2* (*P* = 0.127). Our finding of the association of *NOTCH3* high copy number gain and tumor recurrence fits with many lines of evidence supporting its role as a potent oncogene [9]. The amplification of the region encompassing *NOTCH3* (19p13.12) and upregulation of *NOTCH3* expression has been detected in a high percentage of HGSCs [40]. The upregulation of the NOTCH3 pathway is associated with tumor progression, drug resistance, and HGSC tumor recurrence [9, 41, 42] suggesting that NOTCH3 inhibitors could be an effective treatment in order to increase sensitivity to platinum-based therapies. There also is growing evidence that *PIK3R2*, encoding the p85β regulatory subunit of PI3K, is an oncogene. PIK3R2 induces oncogenic signaling in HGSC [43] and high expression is correlated with a significant reduction in overall survival in ovarian cancer patients [44].

The significance of the high copy gain in *ZNF536* is more difficult to explain as it has not been associated with cancer. However, it may be due to its location near (within 500 kbp) of *CCNE1* at 19q12. The most significantly amplified region from our GISTIC analysis was 19q12. Focal amplification of *CCNE1* is a hallmark of HGSC and is associated with chemoresistance [9, 16, 17, 32]. Interestingly, our Firth’s Cox regression analysis of high-amplitude genes significantly associated with relapse-free survival showed that *ZNF536* was highly significant (*p* = 0.014, Table 2) while *CCNE1* was not significant (*p* = 0.22, Table S6). High-level amplification was more frequent in *CCNE1* (11 of 61 patients) than in *ZNF536* (6 of 61 patients), indicating that *CCNE1* amplification may be an earlier amplification event more related to occurrence of HGSC, rather than outcome after therapy.

Knijnenburg and colleagues calculated homologous recombination deficiency (HRD) scores for 33 cancer types from TCGA and found that HGSC had the highest HRD score and that a higher score within HGSCs was associated with better survival [45] likely due to better response to platinum-based chemotherapies. Although we could not calculate HRD scores, one third of our HGSC patients carried germline or somatic LOF variants in core HRR genes. Compared to patients not carrying these LOF variants, patients with these variants showed significantly better relapse-free survival (*p* < 0.05; Supplemental Fig. 2). In both our tumor samples and TCGA tumor samples, LOF variants in HRR genes were associated with increased incidence of high-amplitude SCNAs at multiple chromosome regions (Supplemental Table 6). This observation suggests that LOF variants in HRR genes caused HRD resulting in greater genomic instability as manifest by increased SCNAs.

HGSC tumors are known for the ability to acquire resistance to the killing effects of various chemotherapeutic agents such as platinum and PARP inhibitors. Platinum resistance can develop from multiple mechanisms including reduced intracellular drug accumulation, intracellular inactivation of the agent, increased DNA repair, or impaired apoptotic signaling pathways [46]. The near ubiquitous somatic mutation of *TP53* is likely the key mechanism in which HGSC tumors evade the triggering of apoptosis.

A strength of this study is the single institution experience with detailed treatment and long-term follow-up data on recurrence and survival. Other strengths are the combined data on germline and tumor DNA sequencing, the OncoScan assay for copy number and large rearrangements, and the availability of multiple tissue sites and/or recurrence debulking tissue for a subset of patients. The primary weakness of the study is the small sample size and that not all samples had the SCNA analysis. Because of limited power, we only ran analyses of LOF variants in *BRCA1, BRCA2*, and *PALB2* with relapse-free and overall survival, as well as genes in amplified regions from tumor analysis. A second weakness is that because we used a targeted gene approach, we did not have the data to generate an HRD score. Lastly, the impact of the genomic alterations on the overall prognosis (relapse-free survival and overall survival) did not account for the use of bevacizumab or PARP inhibitors.

## Conclusion

In summary, from 71 patients with HGCS, we performed targeted germline and tumor sequencing of genes involved in DNA damage response and PI3K/AKT/mTOR pathways and provided a comprehensive analysis of these 557 genes. We identified germline and somatic genetic alterations including SCNAs and analyzed their associations with relapse-free and overall survival. We validated our results using TCGA data. Amplifications in *NOTCH3* and *PIK3R2* were significantly associated with shorter relapse-free survival likely because of over-expression where targeted inhibition may be useful. Interestingly, we identified few changes in mutation profiles from primary debulking versus second debulking samples or from the primary site to metastatic sites from HGSC patients with multiple samples. However, we did not investigate SCNAs in multiple tissues from the same individuals. In conclusion, our single-site long-term follow-up study of HGSC patients was consistent with other studies of HGSC. Our findings suggest that targeted treatments based on both variant and SCNA profile potentially could improve relapse-free and overall survival.

## Methods

### Study subjects

Participants were 71 women with Stage III or IV HGSC who underwent their first debulking surgery between 2002 and 2014 and provided informed consent approved by the City of Hope Institutional Review Board (IRB# 12,358). Subjects were not selected for age or family history. Clinical information was abstracted from medical records and follow-up was through 06/2021. Of the 71 patients, tumor tissue was sequenced from the first debulking surgery for 69 patients; 63 tumors were from the primary site and 6 were from metastatic sites (four tumors in the omentum and two in the pelvis) as no sample was available from the primary site. For two patients, tumor tissue only was available from the second debulking surgery. For 15 patients, we had tumor tissue from both the primary and second debulking surgeries and for 3 patients, we had tumor tissue from 3 surgeries. Twelve patients had multiple tumor sites from the same primary surgery. In total, there were 114 tumor sites sequenced for the 71 patients (details in Supplemental Table 1).

The majority of tumor samples were from banked formalin-fixed-paraffin-embedded (FFPE) sections (*n* = 67). For 47 tumor samples from 22 patients, we obtained fresh samples at the time of surgical debulking and stored tissues in RNAlater solution (ThermoFisher Scientific, Waltham, MA) at -80 °C. Germline DNA was extracted from peripheral blood mononuclear cells using a phenol/chloroform protocol and tumor DNA was extracted from fresh frozen samples or from FFPE sections using the QIAamp DNA Tissue Kit (Qiagen, Hilden, Germany) (Supplemental Table 1).

### High-throughput sequencing and variant calling

We used KAPA Hyper Prep Kits (Kapa Biosystems, Wilmington, MA) to create barcoded paired-end libraries with 300-bp inserts and hybridized the bar-coded samples to a custom NimbleGen SeqCap (Roche, Basel, Switzerland) or SureSelectXT (Agilent, Santa Clara, CA) targeted-gene capture kit. We used custom bait designs which included 557 candidate cancer susceptibility genes involved in the response to DNA damage, DNA repair, cell cycle regulation, programmed cell death, and PI3K/AKT/mTOR pathways (Supplemental Table 2). Following capture, samples were sequenced with 2 × 100 bp paired end reads on a GAIIx or HiSeq 2500 (Illumina, San Diego, CA) in the COH Integrative Genomics Core (IGC) with germline samples sequenced to an average coverage of 84.1-fold and tumor samples sequenced to an average coverage of 84.5-fold. Paired-end reads from each sample were aligned to human reference genome (GRCh37/hg19) using the Burrows-Wheeler Alignment Tool (BWA, v0.7.5a-r405) under default settings [47], and the aligned binary alignment map (BAM) sequence files were sorted and indexed using SAMtools (v0.1.19) [48]. The sorted and indexed BAM files were processed by Picard MarkDuplicates (v1.105, http://picard.sourceforge.net/) to remove duplicate sequencing reads. A pileup file was then created via mpileup in SAMtools [48] and germline and somatic variants were called using Varscan2 (v.2.2.8) [49]. These variants were subsequently annotated using ANNOVAR [50].

### Germline variant filtering

We evaluated variants using Ingenuity Variant Analysis (IVA) Qiagen Clinical Insight version 1.2 (Qiagen Inc, Alameda, CA). IVA used the following content versions: Ingenuity Knowledge Base (X-release), gnomAD (v2.1.1) [51], PhyloP [52], Sorting Intolerant from Tolerant (SIFT) [53], the Human Gene Mutation Database (HGMD, 2019.3), Catalogue of Somatic Mutations in Cancer (COSMIC, v89) [24], and Clinvar (2019–11-06) [54]. Variants with a frequency greater than 2% in the gnomAD database were removed. All remaining variants were visually checked using the Integrative Genomics Viewer [55] to confirm that there were no sequencing errors.

We applied American College of Medical Genetics and Genomics (ACMGG) guidelines to the variants using the ACMGG calling algorithm in IVA [56]. IVA categorizes variants based on standard ACMGG variant calling recommendations (i.e., PVS1, BS2, etc.) by searching available databases and literature for known information for each variant in addition to running in silico models as described above. All ACMGG-called pathogenic (P) or likely pathogenic (LP) variants, as well as protein-truncating variants of unknown significance (PTVUS) were individually evaluated using the available literature and ClinVar to make a final call [54]. PTVUS include frameshift variants, stop codon changes, or variants that disrupt a splice site up to two bases into the intron. For simplicity, we categorized all P, LP, and PTVUS variants as loss-of-function (LOF) variants.

### Assay to test for Hispanic/Latina (H/L) germline founder *BRCA1* exon 9–12 deletion

The 11 H/L patients in our study were tested for the germline founder *BRCA1* exon 9–12 deletion [57]. The deletion was detected by PCR amplification of the mutant and wild type alleles, using specific primers based on the Weitzel et al. method [57]. The PCR products were resolved on a 1.5% agarose gel to identify the amplification of the truncated and wild-type alleles.

### OncoScan assay

OncoScan was performed in the Cytogenetics Core on DNA from 46 FFPE and 15 fresh-frozen tissues from 61 of the 71 patients (Supplemental Table 1). Sufficient DNA samples were not available for tumors from 10 patients for the assay. For the assay, 75 ng of DNA was used for the Molecular Inversion Probe (MIP) amplification, labeling, and hybridization steps (Thermo Fisher Scientific/Affymetrix, Santa Clara, CA). The CEL files (raw data) were then input into Chromosome Analysis Suite (ChAS) software (Thermo Fisher Scientific/Affymetrix, Santa Clara, CA) for processing and normalization with reference files specific for FFPE or fresh tissue. The generated OSCHP files were then loaded to the Nexus Express software (BioDiscovery, El Segundo, CA) to generate per-sample segmentation using the TuScan (Tumor Scan) algorithm developed by Affymetrix.

### Determination of loss of heterozygosity (LOH)

We used the output from the OncoScan assay [58] to assess LOH on DNA from the 61 tumors (Supplemental Table 1). For the 10 patients in which we did not have OncoScan data, we used the variant allele frequencies from tumor sequence to determine LOH (> 20% increase of variant allele frequency over normal was used for defining LOH). For regions demonstrating LOH where there was targeted sequencing data, we confirmed that those demonstrating LOH from OncoScan data also demonstrated LOH in the sequencing data.

### Identification of significant SCNAs

The segmentation files served as input files for the GISTIC2.0 program [39] on the gene pattern server (https://genepattern.broadinstitute.org/gp) to identify significant SCNAs using a q-value cutoff < 0.05. The GISTIC “All Lesion File,” which includes changes in copy number for each SCNA (row) and sample (column), was used as an input matrix for the *hclust* function in R [59] to perform unsupervised hierarchical clustering analysis with Pearson correlation coefficient as a distance metric and a complete linkage method to measure closeness between two clusters.

### Association analysis between LOF variants in core HRR pathway genes and incidence of high-amplitude SCNAs

We tested the association between LOF variants in *BRCA1*, *BRCA2*, and *PALB2* and incidence of high-amplitude SCNAs measured by the OncoScan assay using Firth’s logistic regression model. In the model, high-amplitude SCNAs (yes or no in a tumor sample with a GISTIC2-threshold score of 2 or -2 for a specific gene) was the outcome variable and LOF variant (yes or no in a tumor sample with a germline or somatic LOF variant in at least one core HRR pathway gene) was the predictor variable. This association test was performed only for genes with high-amplitude SCNAs in at least 5% of tumor samples (reducing the effect of rare SCNA events).

*Pathway analysis of genes with significant association between LOF variants in core HRR pathway genes and incidence of high-amplitude SCNAs.* The list of genes with statistically significant associations (*P* < 0.05 and FDR < 0.25) from the Firth’s logistic regression served as input for functional interpretation of the gene list using the g:Profile webserver (http://biit.cs.ut.ee/gprofiler/) and pathway enrichment analysis using Qiagen Ingenuity Pathway analysis (https://digitalinsights.qiagen.com/).

### Analyses of effect of LOF variants in HRR core genes on relapse-free and overall survival

We conducted Kaplan–Meier analyses and log-rank tests to assess differences in relapse-free and overall survival between women carrying LOF variants (germline or somatic) in *BRCA1, BRCA2*, and *PALB2* and those without variants in these genes. Relapse-free survival was defined as time from date of last chemotherapy treatment to cancer recurrence. For relapse-free survival, time was censored at death if the cause of death was not from ovarian cancer or at last contact if the patient was still alive at last contact date.

### Analyses of effect of high-amplitude SCNAs on relapse-free and overall survival

Because cancer driver SCNAs tend to be shorter in length and higher in amplitude than passenger SCNAs [39], we only analyzed high-amplitude SCNAs defined as GISTIC-threshold scores of 2 (high-level amplification of > 4 copies) and -2 (homozygous deletion) [11]. A multivariate Cox regression model was used to assess the association between high-amplitude SCNAs and relapse-free and overall survival, adjusting for age at diagnosis, tumor stage, surgery (optimal vs. suboptimal debulking), type of adjuvant chemotherapy (intra-venous vs. intra-peritoneal), and SCNA rate (the number of SCNAs per sample generated by the Nexus Express software).

TCGA SCNA, variant, and clinical data for late-stage (3 or 4) HGSC patients (*n* = 356) [9] were downloaded from the cBioPortal website (http://www.cbioportal.org) [60]. Because the COH HGSC patients were all stage III and IV tumors, we only included data from TCGA of HGSC with stage III or IV tumors (356/489). These data were used for validation testing of results.

## Supplementary Information

**Additional file 1.**

**Additional file 2.**

## Data Availability

The data sets supporting the results of this article are available and can be accessed in NCBI (National Center for Biotechnology Information) dbGAP (database of Genotypes and Phenotypes) with accession number phs003198.v1.p1.

## Abbreviations

ACMGG: American College of Medical Genetics and Genomics
BAM: Binary alignment map
BWA: Burrows-Wheeler Alignment
ChAS: Chromosome Analysis Suite
COH: City of Hope
FDR: False-discovery rate
FFPE: Formalin-fixed-paraffin-embedded
HGSC: High-grade serous ovarian cancer
H/L: Hispanic/Latina
HRR: Homologous recombination repair
ICL: Interstrand cross links
IGC: Integrative Genomics Core
JNK: C-Jun N-terminal kinase
LOF: Loss-of-function
LOH: Loss of heterozygosity
LP: Likely pathogenic
P: Pathogenic
PTVUS: Protein-truncating variants of unknown significance
SCNA: Somatic copy number alterations
TCGA: The Cancer Genome Atlas
TuScan: Tumor Scan
