# Supplementary material for: Genomic analyses of germline and somatic variation in high-grade serous ovarian cancer
Source: J Ovarian Res. 2023 Jul 17;16:141. doi: 10.1186/s13048-023-01234-x (PMC10351177; doi:10.1186/s13048-023-01234-x)
Supplement: Supplementary file 1 — Additional file 1. [file 13048_2023_1234_MOESM1_ESM.pdf]

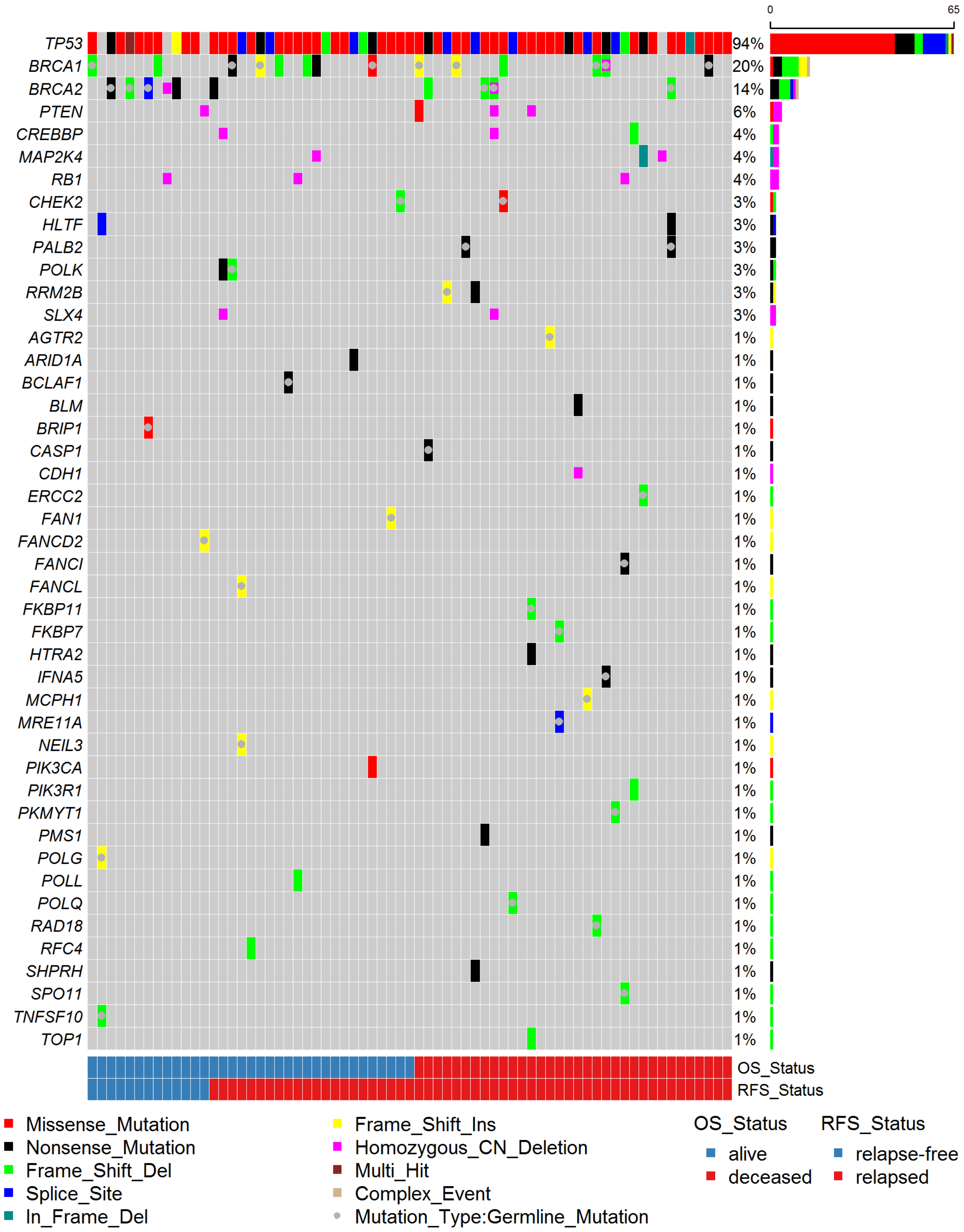

Supplemental Figure 1. Oncoplot of LOF germline mutations and non-silent somatic mutations. OS = overall survival. RFS = relapse-free survival.

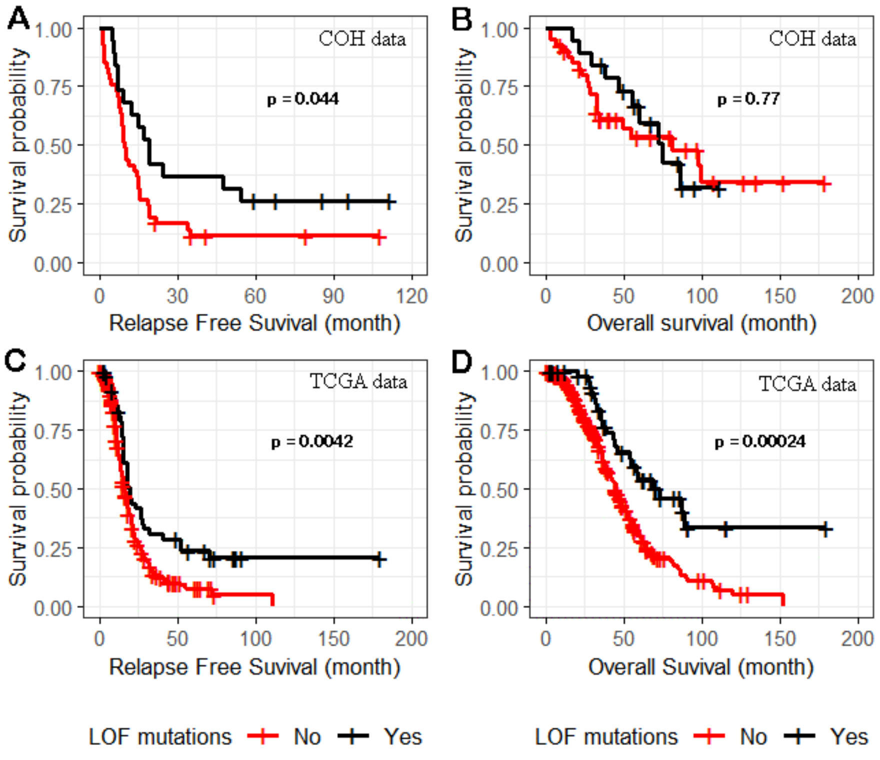

Supplemental Figure 2. Relapse-free and overall survival related to Loss of Function (LOF) mutations in *BRCA1*, *BRCA2*, and *PALB2*. Compared to patients not carrying LOF mutations in any of the three genes, 19 of 61 COH (panel A) and 51 of 367 TCGA patients (panel C) carrying LOF mutations showed significantly ($p < 0.05$) longer relapse-free survival. For overall survival, in TCGA data, those with LOF mutations had significantly longer survival (panel D), which was not observed in COH patients (panel B).

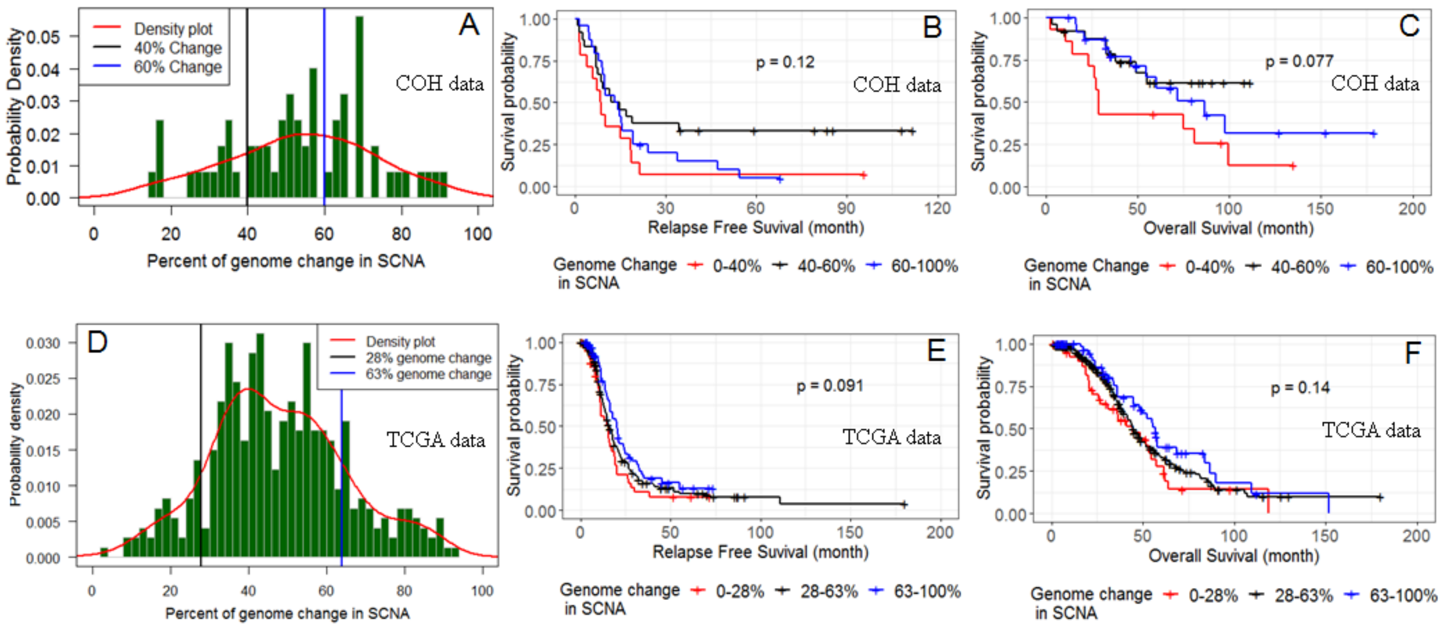

Supplemental Figure 3. Association of percentage of genome changes in SCNAs with relapse-free and overall survival. Histogram, relapse-free survival curves, and overall survival curves in panels A, B, C are for SCNA data of 61 COH patients, respectively, and in panels D, E and F are for data of 367 TCGA patients.
